# Bioinformatic profiling identifies prognosis-related genes in the immune microenvironment of endometrial carcinoma

**DOI:** 10.1038/s41598-021-92091-5

**Published:** 2021-06-15

**Authors:** Pu Cheng, Jiong Ma, Xia Zheng, Chunxia Zhou, Xuejun Chen

**Affiliations:** 1 Department of Gynecology, Second Affiliated Hospital, Zhejiang University School of Medicine, Hangzhou, China; 2 Key Laboratory of Tumor Microenvironment and Immune Therapy of Zhejiang Province, Hangzhou, China

**Keywords:** Cancer, Computational biology and bioinformatics, Oncology

## Abstract

Endometrial carcinoma (EC) is a common malignancy of female genital system which exhibits a unique immune profile. It is a promising strategy to quantify immune patterns of EC for predicting prognosis and therapeutic efficiency. Here, we attempted to identify the possible immune microenvironment-related prognostic markers of EC. We obtained the RNA sequencing and corresponding clinical data of EC from TCGA database. Then, 3 immune scores based on the Estimation of STromal and Immune cells in MAlignant Tumor tissues using Expression data (ESTIMATE) algorithm were computed. Correlation between above ESTIMATE scores and other immune-related scores, molecular subtypes, prognosis, and gene mutation status (including BRCA and TP53) were further analyzed. Afterwards, gene modules associated with the ESTIMATE scores were screened out through hierarchical clustering analysis and weighted gene co-expression network analysis (WGCNA). Differentially expressed analysis was performed and genes shared by the most relevant modules were found out. KEGG pathway enrichment analysis was conducted to explore the biological functions of those genes. Survival analysis was carried out to identify prognostic immune-related genes and GSE17025 database was further used to confirm the correlation between immune-related genes and the ImmuneScore. The immune-related scores based on ESTIMATE algorithm was closely related to the immune microenvironment of EC. 3 gene modules that had the closest correlations with 3 ESTIMATE scores were obtained. 109 immune-related genes were preliminarily found out and 29 pathways were significantly enriched, most of which were associated with immune response. Univariate survival analysis revealed that there were 14 genes positively associated with both OS and PFS. Among which, 11 genes showed marked correlations with ImmuneScore values in GSE17025 database. Our current study profiled the immune status and identified 14 novel immune-related prognostic biomarkers for EC. Our findings may help to investigate the complicated tumor microenvironment and develop novel individualized therapeutic targets for EC.

## Introduction

The global morbidity and mortality of endometrial carcinoma (EC) shows an increasing trend in recent years^1,2^. In China, EC ranks the second place in terms of morbidity among all malignant tumors of female genital system, for whom the 5-year survival rate is 55.1%^3^. At present, FIGO staging and histological classification are still the chief factors applied to patient stratification and prognosis prediction in EC^4,5^. Over the past few past decades, great individual differences have been found in the outcomes of EC treatments due to the tumor heterogeneity, which is partially dependent on the molecular biological features of the primary lesion^6^. Moreover, quite a few patients show distinct responses to adjuvant therapy even though they are at the same clinical stage^7^. Thus, more effective approaches are needed in order to hierarchically classify patients into high- or low- risk subgroups for monitoring and optimizing the treatment of EC^8^.

As in many other types of cancer, immunotherapy is currently recognized as a novel promising therapeutic option in EC^9^. Interactions between different infiltrating immune cells and EC cells in tumor microenvironment (TME) significantly affect tumor progression and recurrence^10^. Besides, EC exhibits a unique immune profile and can be used to construct suitable models for exploring molecular crosstalk between immune and tumor cells^11^. Great progresses have been attained in the past few years, making it possible to recognize novel molecular therapeutic targets within the microenvironment of EC^12^. At the same time, several immune-related factors have been identified to predict patient prognosis, which emphasizes the significance of certain immune status on EC outcomes^13^. Nonetheless, the vast majority of existing studies are pre-clinical basic experiments or have limited available clinical information, while study with a large sample size has not been carried out so far^14^.

Consequently, several immune scoring systems were developed based on the immune-related gene expression patterns through integrated analysis of The Cancer Genome Atlas (TCGA) database to explore the relationships between tumor cells and immunocytes in TME, as well as to quantify the immune microenvironment for each individual cancer case. Of them, the inflammation-based index was reported to be associated with the local immune response and prognosis in various cancers, including pancreatic cancer, colorectal cancer^15^, non-small cell lung cancer^16^ and tongue cancer^17^ and so on. However, limited studies have been designed in an attempt to develop an immune-related prognostic signature for EC.

In the current study, we obtained the RNA sequencing (RNA-seq) and corresponding clinical data of EC from TCGA database. Then, we calculated 3 immune scores for each EC sample based on the Estimation of STromal and immune cells in MAlignant Tumor tissues using Expression data (ESTIMATE) algorithm and analyzed the correlation between ESTIMATE immune score and molecular features of EC. Thereafter, gene modules associated with the immune scores were identified using weighted gene co-expression network analysis (WGCNA), and finally, 14 novel prognostic immune-related genes were screened out which were further explored in the GSE17025 database.

## Materials and methods

### Data collection and immune score calculation

All the data we used in our study are publicly accessible at TCGA and NCBI GEO (accession number: GSE17025) database. Firstly, GDC application programming interface was utilized to download clinical follow-up, RNA-seq and SNP data. RNA-seq FPKM values were subsequently converted into Transcripts PerKilobase Million (TPM) files. The expression levels of 13 previously published immune metagenes^18^ were calculated as the median of log2-transformed expression levels of clustered genes for each sample. Meanwhile, the infiltration of 6 immune cells (B cells, CD4 + T cells, CD8 + T cells, neutrophils, macrophages and dendritic cells) were calculated and downloaded from the TIMER^19^ (https://cistrome.shinyapps.io/timer/) database. In addition, the ESTIMATEScore, StromalScore and ImmuneScore values for each sample were computed by the ESTIMATE function of R package.

### Assessment of the correlation between ESTIMATE scores and immune status

Correlation analysis was carried out to assess the relevance between 3 ESTIMATE immune scores, expression of 13 immune metagenes and the infiltration status of 6 immune cells. Moreover, the correlation of 3 ESTIMATE immune scores, 13 immune metagenes expression and the infiltration of 6 immune cells were further analyzed among the 4 previously reported EC subtypes respectively.

### The correlation between ESTIMATE scores and patient prognosis

We firstly conducted survival analysis using Kaplan–Meier method with survival function of R package to explore the overall survival (OS) of above mentioned 4 EC subtypes respectively. Afterwards, patients were divided into high- and low-score groups according to the median of 3 ESTIMATE immune scores. Then, the differences of OS between these groups were examined through Kaplan–Meier method with survival package under R environment.

### Exploration of the association between ESTIMATE scores and gene mutation

The mutation data of BRCA2, BRCA1 and TP53 were extracted from TCGA derived SNP dataset and processed with Mutect. Then, the correlation between different mutation status of these genes and ESTIMATE immune scores were analyzed using Wilcox.test Package under R environment. Furthermore, patients were divided into high- and low- tumor mutation burden (TMB) groups according to the median of TMB value. The correlation between TMB status and ESTIMATE immune scores were assessed using Wilcox.test Package under R environment.

### Identifying immune score-related gene modules through WGCNA

Firstly, transcripts that had at least 75% TPM greater than 1 and median absolute deviation (MAD) greater than the median were selected for following analysis according to the expression patterns. Then, samples were clustered through hierarchical clustering method with the distance > 80,000 considered as cut-off value of outlier sample. The distance between each 2 transcripts was computed according to Pearson correlation coefficient, then the R package WGCNA^20^ was used to establish the weight co-expression network, and co-expression modules were selected at the soft threshold of 10 in order to ensure the constructed co-expression network conformed to the scale-free network. That was to say, the node/k connectivity (log(k)) logarithm was negatively correlated with logarithm in terms of the occurrence probability of node (log(P(k)), and the correlation coefficient was > 0.8. The appropriate β value was also selected to ensure the scale-free network. Then, the expression matrix was converted into the adjacent one, followed by further conversion into the topological one for gene clustering on the basis of topological overlap matrix (TOM), in accordance with the average linkage hierarchical cluster approach following the mixed dynamic shear tree standards. In addition, more than 30 genes had been selected for every gene network module. The dynamic shear approach was also utilized to determine gene modules, and eigengenes values of all modules were calculated. Cluster analysis was then carried out on the modules, adjacent modules were fused together to obtain a novel one, and appropriate minModuleSize, deepSplit, and height values were assigned. Kyoto Encyclopedia of Genes and Genomes (KEGG) pathway enrichment analysis was performed to explore the potential biological functions of genes within these 3 modules using the ClusterProfiler package under R environment, with the significant FDR level of < 0.05. Then, the associations between the recognized gene modules and ESTIMATEScore, StromalScore as well as ImmuneScore values were calculated, respectively, in order to mine the gene modules having highest correlation.

### Construction of gene interaction network and functional analyses

We separated patients into two groups equally based on the ImmuneScore and ESTIMATEScore. The DESeq2 function of R package was utilized to screen differentially expressed genes (DEGs) between these groups. The cut-off criterion for DEGs was set as *p* < 0.05 and |log2(Foldchange)|> 1. Genes shared by the most relevant module, DEGs of ImmuneScore and ESTIMATEScore groups were finally screened out. All genes were subsequently mapped into the String database^21^ separately using the STRINGdb package under R environment, with the threshold of > 0.4 to obtain the gene–gene interaction. Cytoscape was used for visualization. At the same time, the R package clusterprofile^22^ was utilized to carry out KEGG enrichment analyses for visualizing the signaling pathways affected by genes. The prognostic value of each immune-related gene was calculated using survival package under R environment. The correlations between immune-related genes and ImmuneScore were further explored with an independent external GEO dataset (GSE17025) through Pearson correlation analysis.

## Results

### The immune scoring system based on the ESTIMATE algorithm was closely related to the immune microenvironment of EC

**Figure 1:**
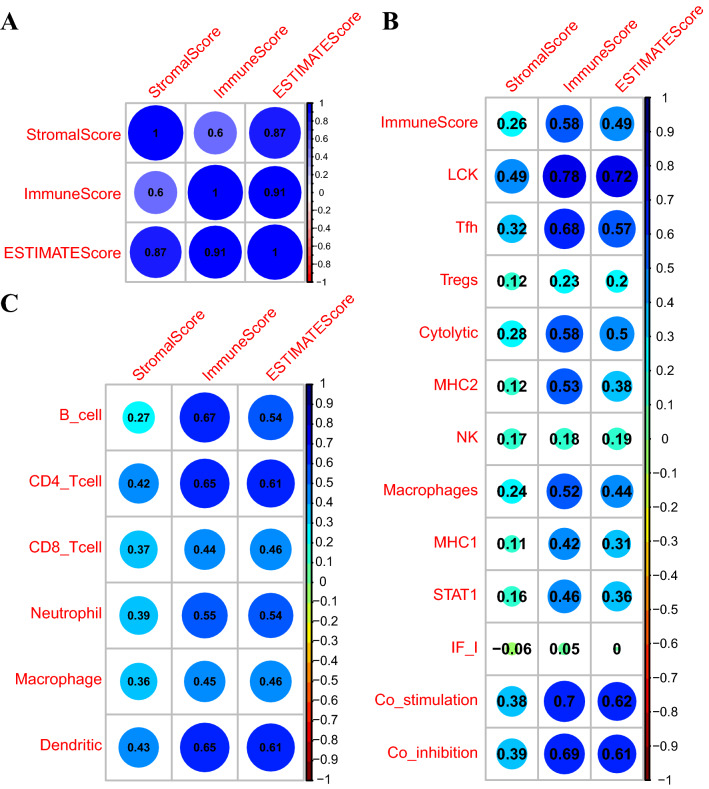
Correlations between ESTIMATE immune score and other immune scores for EC patients. (**A**) Correlations among the 3 ESTIMATE immune scores. (**B**) Correlations between 3 ESTIMATE immune scores and 13 metagenes scores for EC patients. (**C**) Correlations between 3 ESTIMATE immune scores and 6 immunocyte infiltration scores for EC patients. Coefficients of Spearman correlation were displayed color-coded, so as to demonstrate the negative (red) or positive (blue) association.

The expression levels of 13 immune metagenes, infiltration status of 6 types of immunocytes, and 3 immune-related scores based on ESTIMATE algorithm (ESTIMATEScore, StromalScore, and ImmuneScore) were computed. Additionally, Spearman correlation coefficient was employed for quantifying the relationships between these scoring systems (Fig. 1). Our results suggested that, immune-related scores calculated using the ESTIMATE algorithm had an average internal correlation > 0.8 and also high correlations with the other 2 algorithms. Above findings indicated that immune-related scores computed based on the ESTIMATE algorithm was closely related to the immune microenvironment of EC.

**Figure 2:**
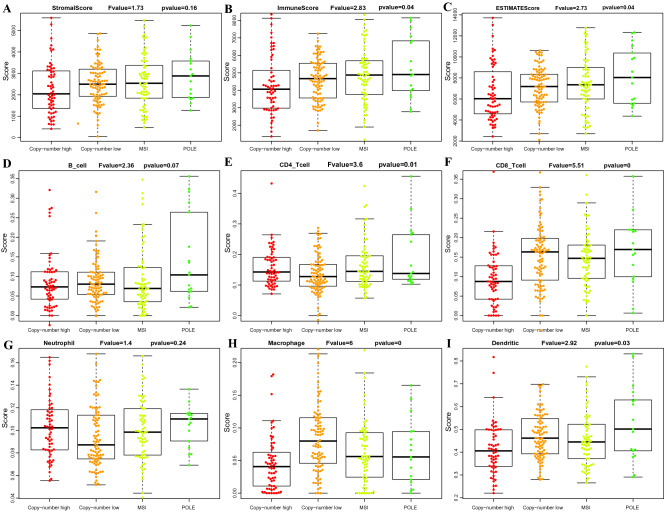
Distribution of StromalScore (**A**), ImmuneScore (**B**), ESTIMATEScore (**C**) and B cell (**D**), CD4 + T cell (**E**), CD8 + T cell (**F**), Neutrophil (**G**), Macrophage (**H**), Dendritic cell (**I**) infiltration among 4 different molecular subtypes (Copy − number high, Copy − number low, MSI and POLE).

Besides, ESTIMATEScore, ImmuneScore, and StromalScore among the 4 recognized subtypes^23^ had also been examined (Fig. 2A–C). It was clear that, differences in ESTIMATEScore and ImmuneScore levels among different molecular subtypes of EC were statistically significant. Moreover, distribution of 6 types of immunocytes infiltration (Fig. 2D–I) and 13 metagenes expression (Fig.S1) among these 4 subtypes was also analyzed. Statistical significances were observed in 6 out of 13 metagenes expression and 5 out of 6 types of immunocytes infiltration.

**Figure 3:**
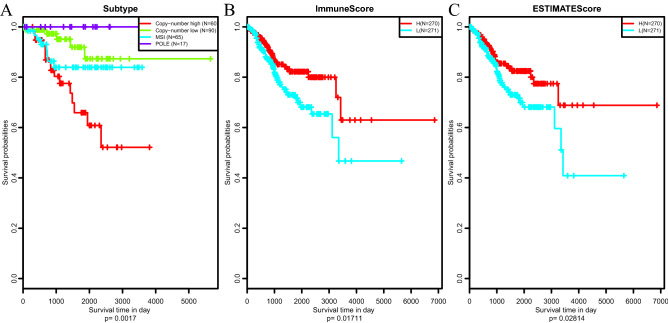
Prognosis of EC patients with different molecular subtypes (**A**), ImmuneScores (**B**) and ESTIMATEScores (**C**). H: high-risk; L: low-risk.

To assess the relationship between 3 ESTIMATE scores and patient prognosis, the prognosis of above 4 subtypes was firstly examined. As shown in Fig. 3A, difference in patient prognosis across those 4 subtypes was statistically significant, among which, the copy − number high subtype had the poorest prognosis. Next, all samples were classified according to median of ESTIMATE scores followed by survival analysis, which was consistent with previous report^24^ (Fig. 3B–C). Obviously, the prognosis for samples with the high ESTIMATEScore and ImmuneScore was much better than that with low scores, suggesting that ESTIMATE immune scoring system might be used as novel promising markers to predict the prognosis for EC.

**Figure 4:**
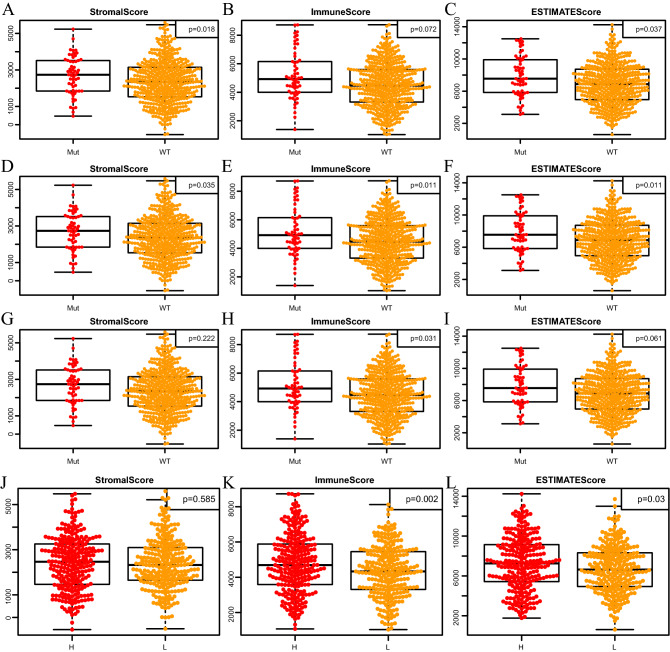
Correlations between ESTIMATE immune scores and gene mutations. The StromalScore in TP53 (**A**), BRCA1 (**D**) and BRCA2 (**G**) non-mutation and mutation groups. ImmuneScore in TP53 (**B**), BRCA1 (**E**) and BRCA2 (**H**) non-mutation and mutation groups. ESTIMATEScore in TP53 (**C**), BRCA1 (**F**) and BRCA2 (**I**) non-mutation and mutation groups. StromalScore (**J**), ImmuneScore (**K**) and ESTIMATEScore (**L**) in TMB-high and -low groups.

Afterwards, correlations between those 3 ESTIMATE scores and 3 independent prognostic gene mutations (BRCA2, BRCA1 and TP53) were explored^25,26^. As a result, 3 immune-related scores of different mutant groups were generally higher when compared with those of wild-type groups, especially in TP53 and BRCA1 subgroups (Fig. 4A–I). Subsequently, the TMB of each sample was computed, and the relationships between 3 ESTIMATE scores and TMB level were analyzed. As presented in Fig. 4J–L, the ESTIMATEScore and ImmuneScore showed significant positive correlation with TMB.

To sum up, the immune-related scores based on ESTIMATE algorithm was closely related to the immune microenvironment of EC and could be used to be the optimal immune scoring methods for prognosis prediction.

### Identification of immune score-related gene modules by WGCNA

**Figure 5:**
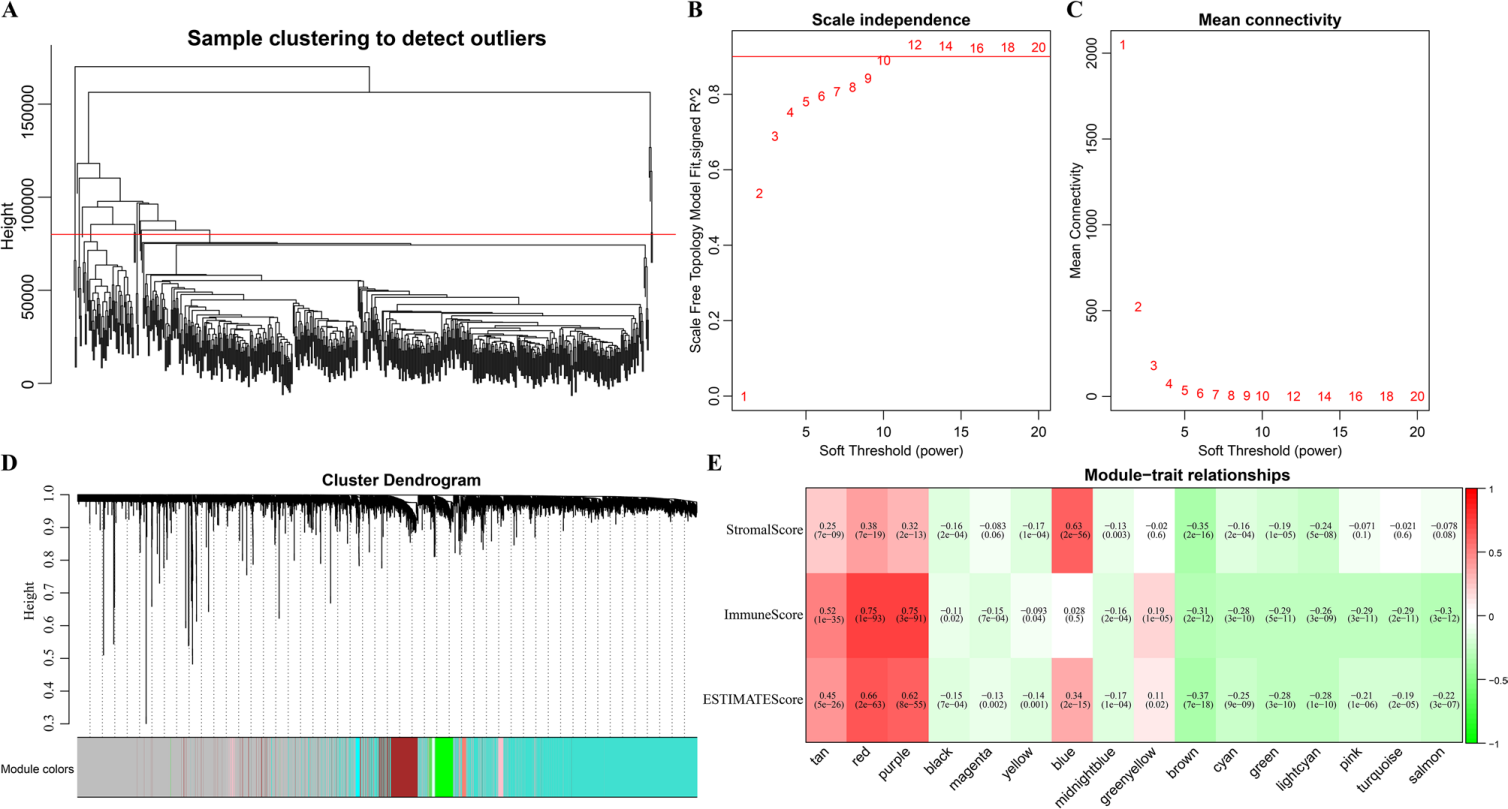
Immune score-related gene modules identified via WGCNA. (**A**) Cluster analysis of samples. (**B**,**C**) Network topological analysis for different soft-thresholding powers. (**D**) Module colors and gene dendrogram. (**E**) Correlations between different gene modules and 3 ESTIMATE Scores. The intensity of red and green colors indicates the value of correlation coefficient.

Samples were firstly selected to performed hierarchical clustering analysis (Fig. 5A). After excluding outlier data, 579 samples were finally used to construct a weight co-expression network through WGCNA (Fig. 5B,C). Then, gene modules were examined by dynamic shear, and all the recognized modules were further clustered. Next, adjacent gene modules were fused to form a new one, with minModuleSize, deepSplit and height set at 30, 2 and 0.25, respectively. 17 modules were ultimately obtained (Fig. 5D,E), and 5362 transcripts were assigned to 16 co-expression modules except the grey module. Correlations between eigenvectors of these 16 modules and 3 ESTIMATE scores were then calculated (Fig. 5E), from which we could see that the red (170 genes), purple (55 genes) and tan (49 genes) modules had the closest correlations with 3 ESTIMATE scores, with the average correlation coefficients > 0.5.

**Figure 6:**
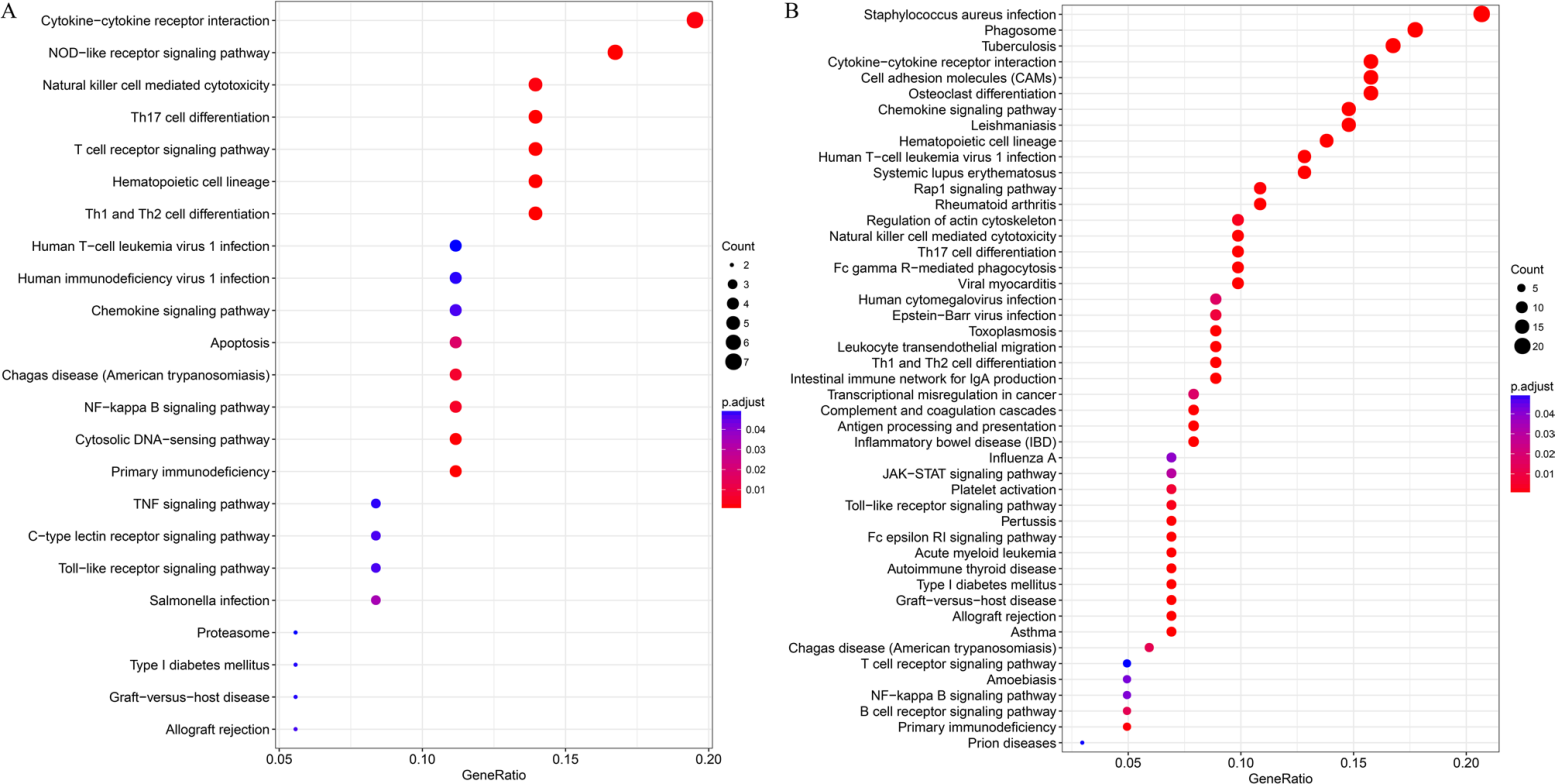
The KEGG pathway enrichment analysis of the genes in purple (**A**) and red (**B**) module.

As following, we performed KEGG pathway enrichment analysis to explore the potential biological functions of the 274 genes within these 3 modules. According to the results, there were 23 and 54 pathways enriched to the purple module and red module, respectively (Fig. 6). It was easy to find that the genes were primary enriched to immune-related pathways, including T cell differentiation, primary immunodeficiency, chemokine signaling pathway, cytokine-cytokine receptor interaction, B cell receptor signaling pathway and so on. Thus, it can be inferred that the ESTIMATE score-related genes may closely associate with the immune microenvironment of EC.

### Identification of immune microenvironment-related prognostic genes

**Figure 7:**
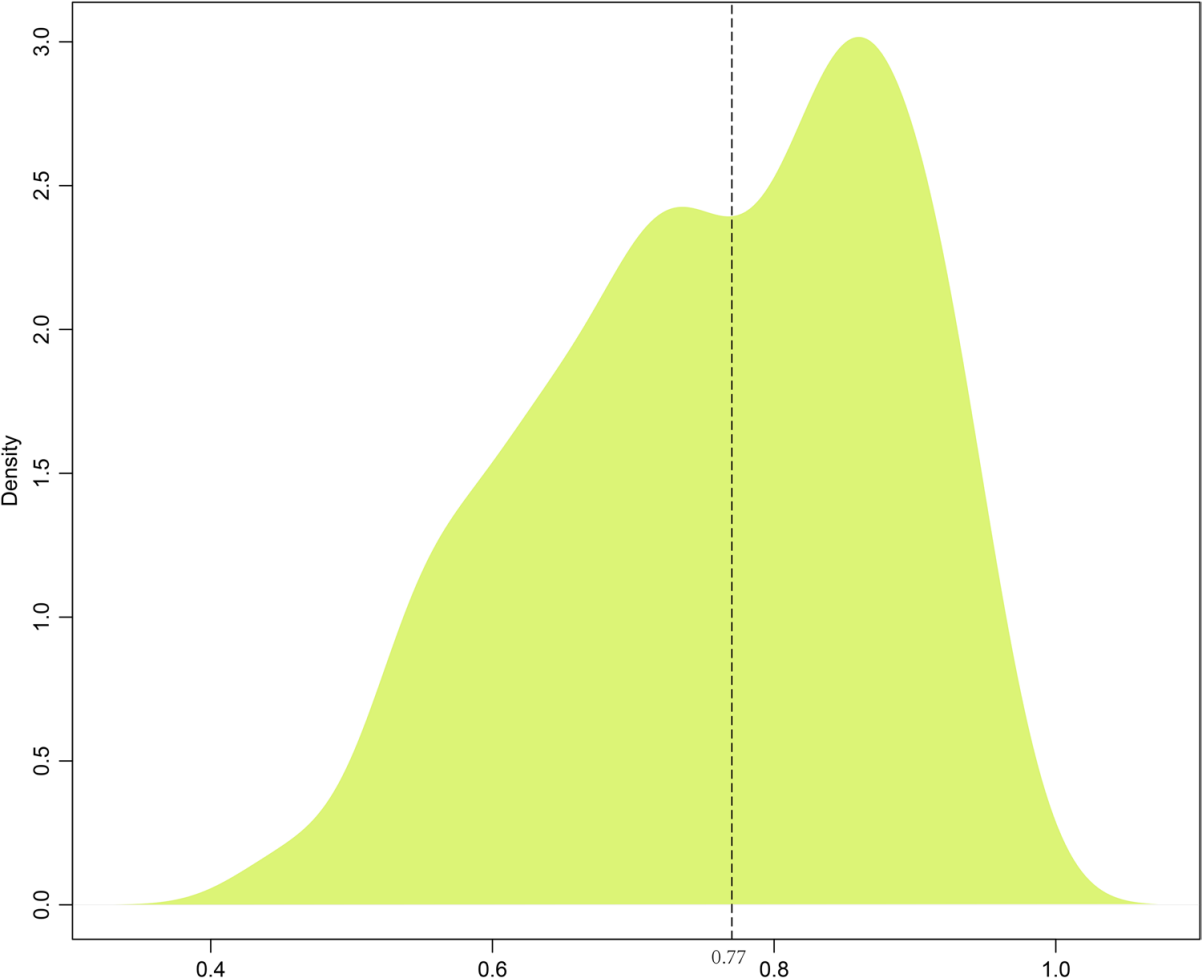
The distribution of maximum correlation coefficients of 274 module-related genes.

To seek out the most immune-related genes, correlations between previously obtained ESTIMATE score-related modules and genes were computed. As illustrated in Fig. 7, the correlation coefficients were distributed in a bimodal manner, with the intersection point value of 0.77. Based on this, 136 genes with the maximum correlation coefficients with those 3 modules greater than 0.77 were screened out.

**Figure 8:**
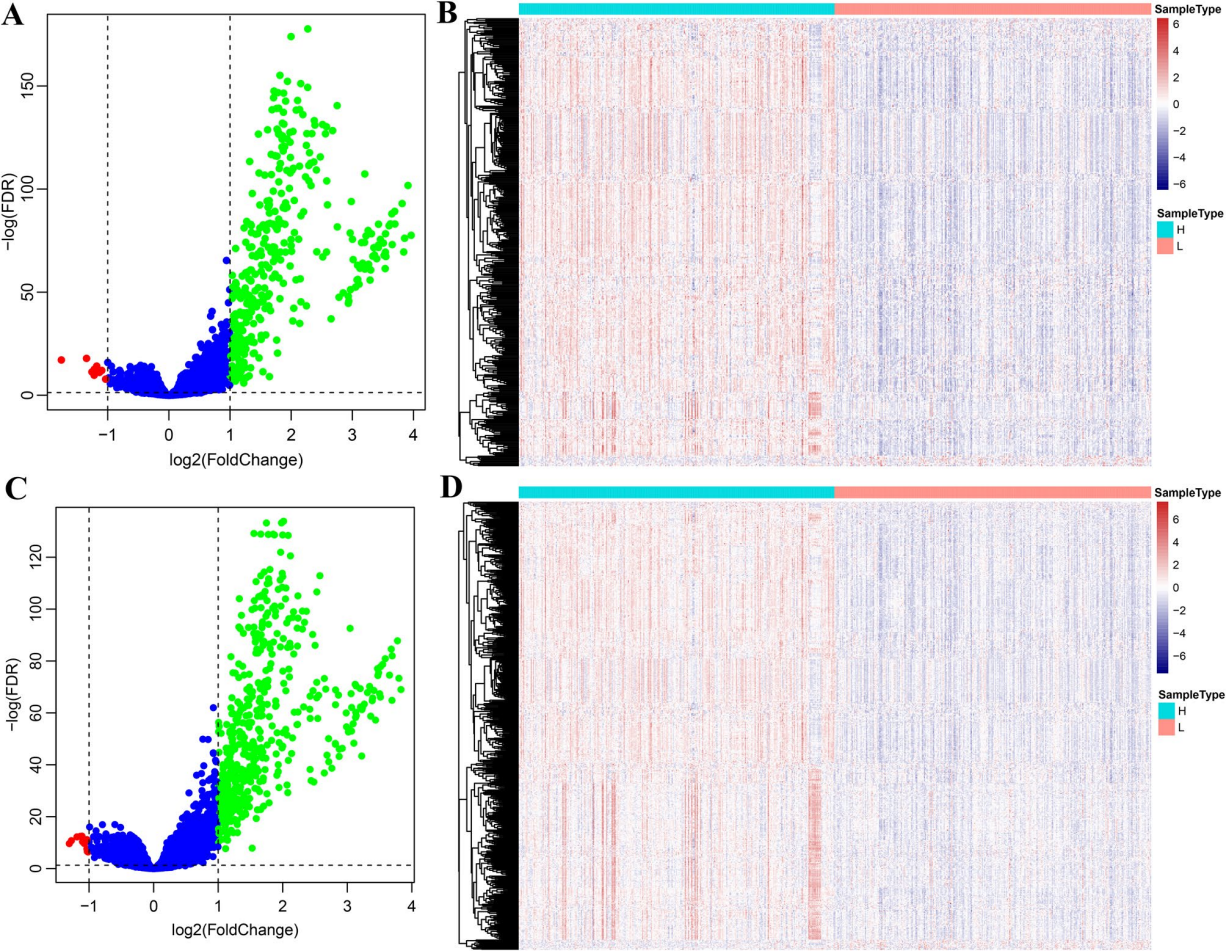
Gene expression profile of high- and low-ImmuneScore /ESTIMATEScore groups. (**A**,**C**) Volcanic maps. (**B**,**D**) Heatmap.

According to the previously described screening method, 379 DEGs were identified among high- and low-ESTIMATEScore subgroups (Fig. 8A,B) using the R package DESeq2^27^. At the meantime, 526 DEGs were acquired among ImmuneScore-high and -low subgroups (Fig. 8C,D). The results were presented in a volcano plot and a heatmap for each subgroup. Obviously, the DEGs showed distinct expression patterns in low-ESTIMATEScore subgroup compared with high-ESTIMATEScore subgroup. Similar results were also observed in low- and high-ImmuneScore subgroups.

**Figure 9:**
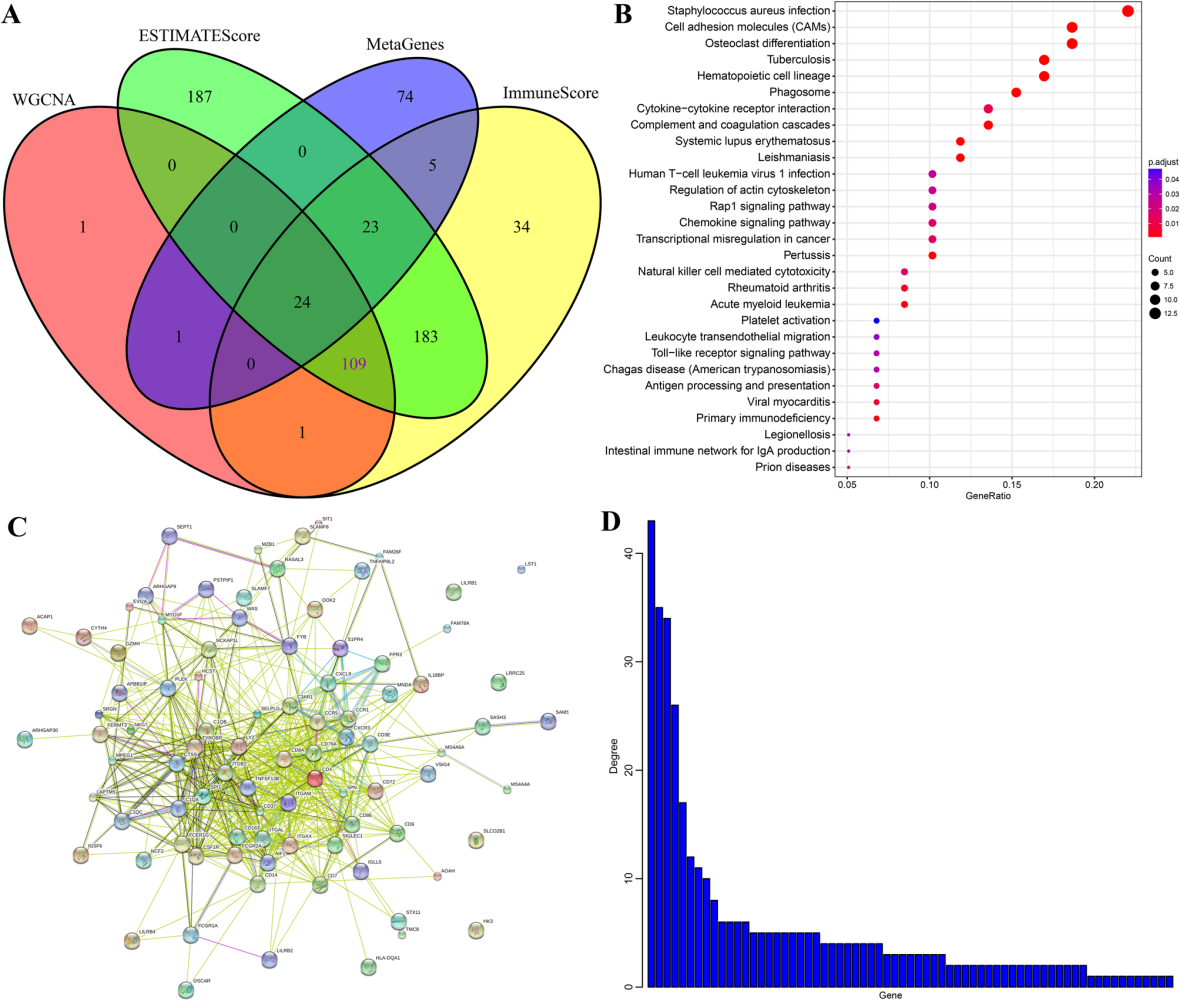
Mining of the immune-related genes. (**A**) The co-expressed genes that were markedly correlated with ImmuneScore/ESTIMATEScore. (**B**) KEGG enrichment analysis of the 109 genes. (**C**) Protein–protein interaction network of the 109 genes. (**D**) The degree value of each node within protein–protein interaction network.

To further investigate the immune-related genes, we integrated above 3 gene sets (136 genes with the maximum correlation coefficients with 3 modules, 379 DEGs in high- and low-ESTIMATEScore subgroups and 526 DEGs in high- and low-ImmuneScore subgroups). After intersection, 133 common genes were obtained and 24 genes belonged to 13 metagenes were eliminated subsequently. Finally, 109 immune-related genes were preliminarily found out (Fig. 9A).

Subsequently, KEGG enrichment analyses was conducted with clusterProfiler package under R environment. As shown in Fig. 9B, 29 pathways were significantly enriched, most of which were associated with immune response. Afterwards, the R STRINGdb package^28^ was utilized to analyze the protein–protein interaction network of those 109 genes. All these genes were mapped to String database in order to acquire the relationship network and 67 nodes were finally obtained (Fig. 9C). As is depicted in Fig. 9D, the degree value of each node was high (5.49 on average), which demonstrated the close association between those immune-related genes.

**Figure 10:**
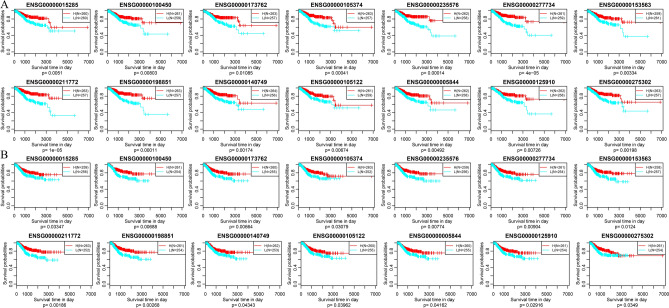
Correlations between 14 novel immune-related genes and OS (**A**) and PFS (**B**) of EC patients. *H* high-expression, *L* low-expression.

Then, univariate survival analysis was performed to identify the immune microenvironment-related prognostic markers using the survival and expression data of above 109 genes. The results revealed that there were 34 and 19 genes associated with OS and PFS, respectively. Among which, 14 genes were found to be positively correlated with both OS and PFS (Table 1, Fig. 10).

**Table 1:** 14 immune-related genes positively correlated with both OS and PFS of EC.

Gene symbol	Gene name	*p* value (OS)	p value (PFS)
WAS	Wiskott-Aldrich snydrome	0.005103711	0.033465715
GZMH	Granzyme H	0.008033252	0.006877271
CD7	CD7 molecule	0.010859632	0.006642321
NKG7	Natural killer cell granule protein 7	0.000412089	0.030792653
LINC01871	Long intergenic non-protein coding RNA 1871	0.000140799	0.007739673
TRAC	T cell receptor alpha constant	4.40E-05	0.009042146
CD8A	CD8a molecule	0.003341004	0.012404753
TRBC2	T cell receptor beta constant	1.03E-05	0.001855756
CD3E	CD3e molecule	0.000106431	0.002676012
IGSF6	Immunoglobulin superfamily menmer 6	0.00173721	0.043427798
RASAL3	RAS protein activator like 3	0.006739461	0.039618006
ITGAL	Inergrin subunit alpha L	0.004923864	0.041623366
S1PR4	Sphingsine-1-phosphate receptor 4	0.007261307	0.029160931
CCL4	C–C motif chemokine ligand 4	0.001976119	0.034895726

### Further exploration of the correlations between 14 immune-related prognostic genes and ImmuneScore using an external database

**Figure 11:**
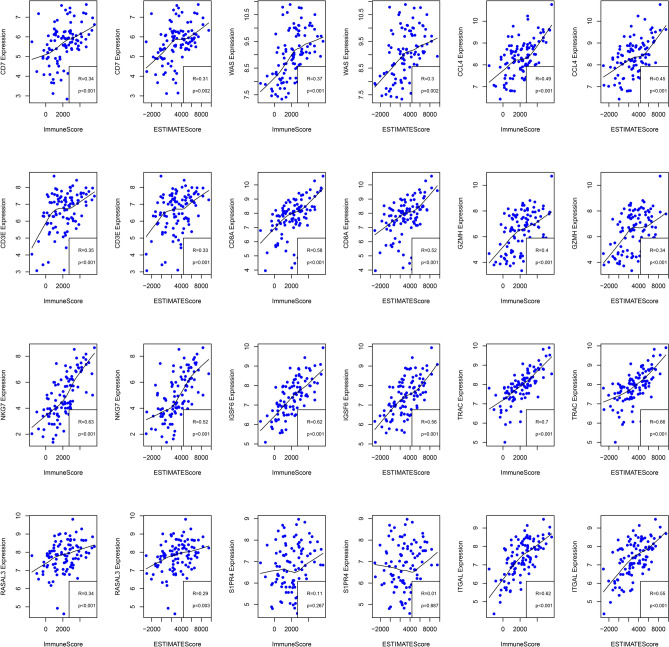
Correlations between 12 immune-related prognostic genes and ImmuneScore/ESTIMATEScore within GSE17025 dataset.

The independent dataset GSE17025^29^ was chosen and normalized expression matrix was downloaded. Then, the R package ESTIMATE was used to calculate the sample ImmuneScore values. Pearson correlation coefficients between expression levels of 12 genes (2/14 genes were excluded due to unavailable expression data) and ImmuneScore values were further determined. As illustrated in Fig. 11, 11 genes (except S1PR4) showed marked correlations with ImmuneScore values.

## Discussion

Until recently, the 5-year survival of advanced or recurrent EC is still not optimistic. Even though postoperative adjuvant therapy (chemotherapy and/or radiotherapy) can improve patient outcome, great individual differences in therapeutic effect exist due to the biological heterogeneity of EC cells. Hence, there is a critical need for reliable prognostic biomarkers to evaluate the risk of cancer progression and develop the patient-tailored treatment strategy.

With the rapid development of next generation sequencing techniques, numbers of novel molecular biomarkers have been identified, which were of great help to the personalized treatment of EC^8,30^. Nevertheless, most studies are conducted on animal models, surgical cancer tissues samples or cell lines under in vitro conditions.

Tumor microenvironment, which is comprised of tumor cells, stromal cells and the secreted inflammatory mediators and cytokines, is a complex system and supports various tumor biological behaviors, including tumor genesis, progression, metastasis and so on. Stromal cells are mainly made up of immunocytes, fibroblasts, mesenchymal cells and tumor-associated endothelial cells. The abnormal infiltration of stromal cells, especially for immunocytes (such as neutrophils, monocytes and lymphocytes), has been verified in numerous studies^11,31^. For now, great attention has been paid to the association between immune system and tumor biology^32^. A growing number of studies have not only revealed the interaction between EC cells and the host immune system, but also enhanced the efficacy of immunotherapies^33^. As mentioned before, EC has been widely accepted as a kind of immunogenic malignancy. The treatment of EC has reached a new milestone through artificially manipulating the tumor immune microenvironment. Therefore, it is of great importance for us to explore the immune-related prognostic and therapeutic biomarkers for EC^34^.

In current study, we used the RNA-seq data downloaded from TCGA database to calculated 3 immune-related scores based on ESTIMATE algorithm, which showed marked correlation with the immune status, survival time, prognosis-related gene mutations, and molecular subtypes of EC. Next, ESTIMATE immune score-related gene modules were obtained by means of WGCNA. 109 immune-related genes were then screened out using differential expression analysis and their functions were examined through enrichment analysis. Survival analysis was then performed and 14 novel immune-related prognostic genes were finally screened out, among which, 12 genes were further confirmed having marked correlations with ImmuneScore values in GSE17025 dataset.

Our newly discovered 14 immune-related prognostic markers include WAS, GZMH, CD7, NKG7, LINC01871, TRAC, CD8A, TRBC2, CD3E, IGSF6, RASAL3, ITGAL, S1PR4 and CCL4. Unfortunately, only a few studies have explored the roles of these genes in EC. Among these, 4 genes (CD8A, CD3E, CCL4 and ITGAL) were reported to have close relationship with tumor immune microenvironment and to be involved in various pathological processes of EC^35–37^. TRBC2 encodes a specific region of the T-cell receptor beta-2 chain^38^ and has been identified as a promising biomarker for the distinction of multiple cancer types, including breast cancer, colorectal cancer, glioblastoma, hepatobiliary cancer, lung cancer and pancreatic cancer and so on^39^. It is worth mentioning that, our findings demonstrated that TRBC2 had the most significant correlation with both OS and PFS of EC patients. Nevertheless, no studies have validated the specific role of TRBC2 in EC yet. Thus, it really deserves further study to elucidate the clinical importance and underlying molecular mechanism of TRBC2 in EC.

## Conclusion

Briefly, our current study focuses on gene features associated with EC immune microenvironment. According to our findings, these genes participate in the progression of EC, and affect patient prognosis. Our work helps to investigate the complicated interactions in EC microenvironment. At the same time, our work may help to develop novel potential prognostic biomarkers and therapeutic targets for EC.

## Supplementary Information

Supplementary Information.

## Data Availability

The data generated are included in the manuscript and supplementary data. All the data we used in our study are publicly accessible at TCGA and NCBI GEO database.

## Abbreviations

EC: Endometrial carcinoma
ESTIMATE: MAlignant tumor tissues using expression data
FIGO: The international federation of gynecology and obstetrics
TME: Tumor microenvironment
TCGA: The cancer genome atlas
WGCNA: Weighted gene co-expression network analysis
GEO: Gene expression omnibus
TPM: Transcripts PerKilobase million
OS: Overall survival
PFS: Progression-free survival
TMB: Tumor mutation burden
MAD: Median absolute deviation
TOM: Topological overlap matrix
KEGG: Kyoto encyclopedia of genes and genomes
DEGs: Differentially expressed genes
