# Supplementary material for: Bioinformatic profiling identifies prognosis-related genes in the immune microenvironment of endometrial carcinoma
Source: Sci Rep. 2021 Jun 15;11:12608. doi: 10.1038/s41598-021-92091-5 (PMC8206132; doi:10.1038/s41598-021-92091-5)
Supplement: Supplementary file 1 — Supplementary Information. [file 41598_2021_92091_MOESM1_ESM.docx]

**Bioinformatic profiling identifies prognosis-related genes in the immune microenvironment of endometrial carcinoma**

Pu Cheng^1,2,*^, Jiong Ma ^1^, Xia Zheng^1^, Chunxia Zhou^1^, Xuejun Chen ^1^

^1^ Department of Gynecology, Second Affiliated Hospital, Zhejiang University School of Medicine, Hangzhou, China

^2^ Key Laboratory of Tumor Microenvironment and Immune Therapy of Zhejiang Province

Pu Cheng, Ph.D. Email: drchengpu@zju.edn.cn

Jiong Ma, MD. Email: majiong@zju.edu.cn

Xia Zheng, MD. Email: xiazheng0506@foxmail.com

Chunxia Zhou, MD. Email: 2515135@zju.edu.cn

Xuejun Chen, Ph.D. Email: 2303011@zju.edu.cn

*** Correspondence:**

Pu Cheng

drchengpu@zju.edn.cn

**
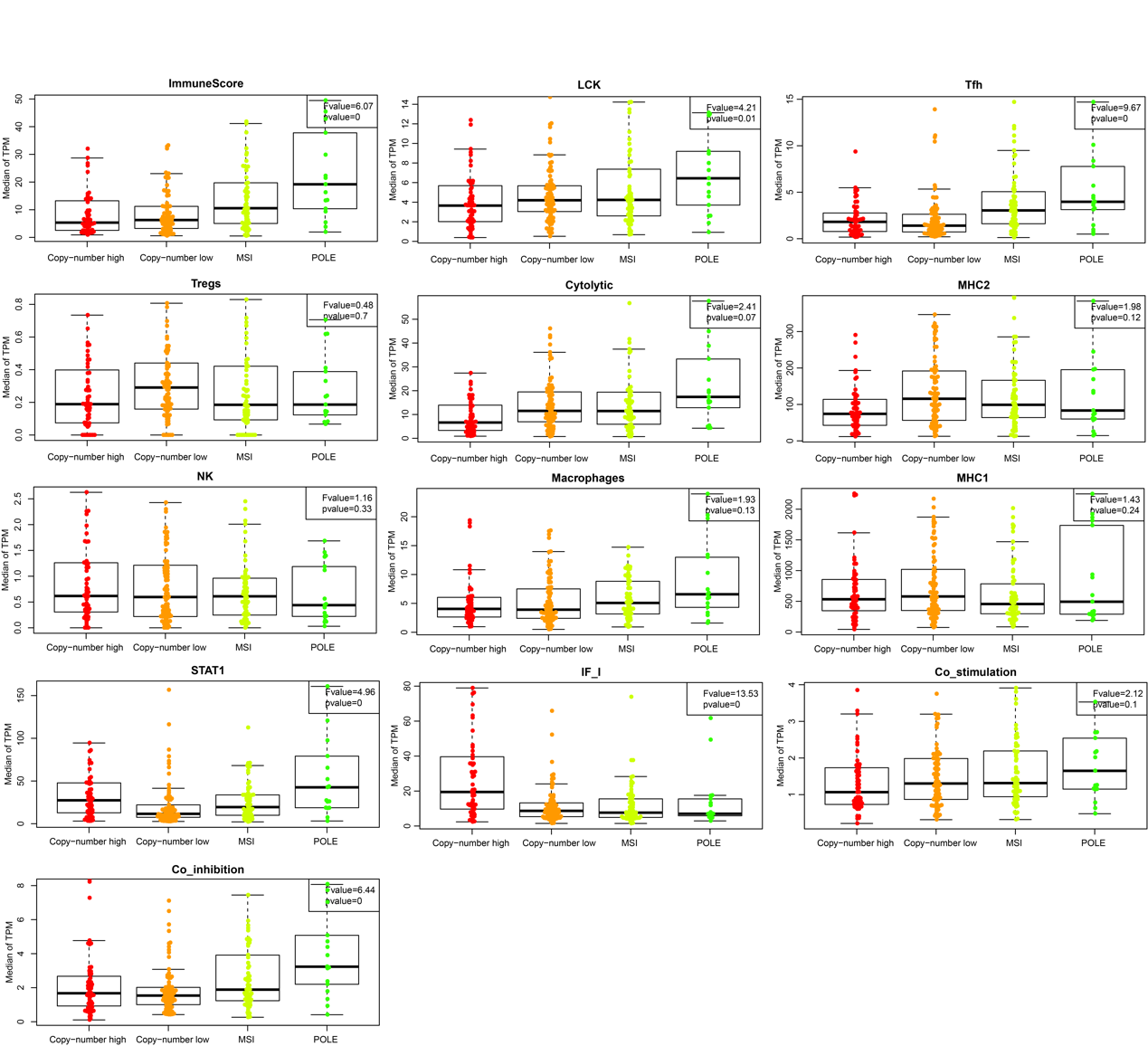
**

**Fig.S1 Distribution of 13 metagenes expression level**
